# Ubiquitin-dependent trafficking and turnover of ionotropic glutamate receptors

**DOI:** 10.3389/fnmol.2015.00060

**Published:** 2015-10-16

**Authors:** Marisa S. Goo, Samantha L. Scudder, Gentry N. Patrick

**Affiliations:** Section of Neurobiology, Division of Biological Sciences, University of CaliforniaSan Diego, La Jolla, CA, USA

**Keywords:** glutamate receptor, ubiquitin, E3 ligase, deubiquitinating enzyme (DUB), proteasome, lysosome, postsynaptic density, synaptic plasticity

## Abstract

Changes in synaptic strength underlie the basis of learning and memory and are controlled, in part, by the insertion or removal of AMPA-type glutamate receptors at the postsynaptic membrane of excitatory synapses. Once internalized, these receptors may be recycled back to the plasma membrane by subunit-specific interactions with other proteins or by post-translational modifications such as phosphorylation. Alternatively, these receptors may be targeted for destruction by multiple degradation pathways in the cell. Ubiquitination, another post-translational modification, has recently emerged as a key signal that regulates the recycling and trafficking of glutamate receptors. In this review, we will discuss recent findings on the role of ubiquitination in the trafficking and turnover of ionotropic glutamate receptors and plasticity of excitatory synapses.

## Introduction

Glutamatergic synapses mediate the majority of excitatory synaptic transmission in the mammalian central nervous system (CNS). Arguably, AMPA receptor (AMPAR) trafficking to and from the postsynaptic membrane plays a significant role in many forms of synaptic plasticity (Shepherd and Huganir, 2007). AMPARs are tetrameric receptors comprised of four different subunits (GluA1-A4) and these subunits can combine in different stoichiometries to form ion channels with distinct functional properties (Hollmann and Heinemann, 1994; Rosenmund et al., 1998). A large body of evidence suggests that AMPARs are not statically localized at the synapse, but rather are dynamically trafficked in and out of the postsynaptic membrane under specific signaling cues.

Phosphorylation is one well-studied post-translational modification that regulates AMPAR trafficking. Protein kinases can phosphorylate AMPARs, which signals them to move to and from the synapse, potentially leading to either long-term potentiation (LTP) or long-term depression (LTD) (Lu and Roche, 2012). Recently, ubiquitination, a distinct post-translational modification, has emerged as an important regulator of AMPAR trafficking and function. Ubiquitin, a 76 amino acid protein, is covalently linked to lysine residues on a protein substrate via an isopeptide bond (Pickart, 2004). The addition of the ubiquitin moiety occurs through a series of enzymatic reactions involving an activating enzyme (E1), a conjugating enzyme (E2), and a ligase (E3) (Mabb and Ehlers, 2010; Berndsen and Wolberger, 2014). Alternatively, removal of the ubiquitin moiety is facilitated by deubiquitinating enzymes (DUBs). Depending on the chain length and topology, the ubiquitin moiety can then send the target protein to various fates in the cell including proteasomal or lysosomal degradation (Pickart and Eddins, 2004). Degradation via the proteasome typically involves a ubiquitin chain length of four or more. On the other hand, ubiquitination in the form of single (mono) or short-chain ubiquitin modifications can result in the endocytosis of integral membrane proteins (Clague and Urbé, 2010). Ubiquitinated proteins are then sorted by the endosome sorting complexes required for transport (ESCRTs) into multivesicular bodies (MVBs) and eventually the lysosome (Hicke and Dunn, 2003; Piper and Luzio, 2007). Conversely, if a DUB acts on the protein in the early endosome, the protein can be recycled back to the plasma membrane.

**Figure 1:**
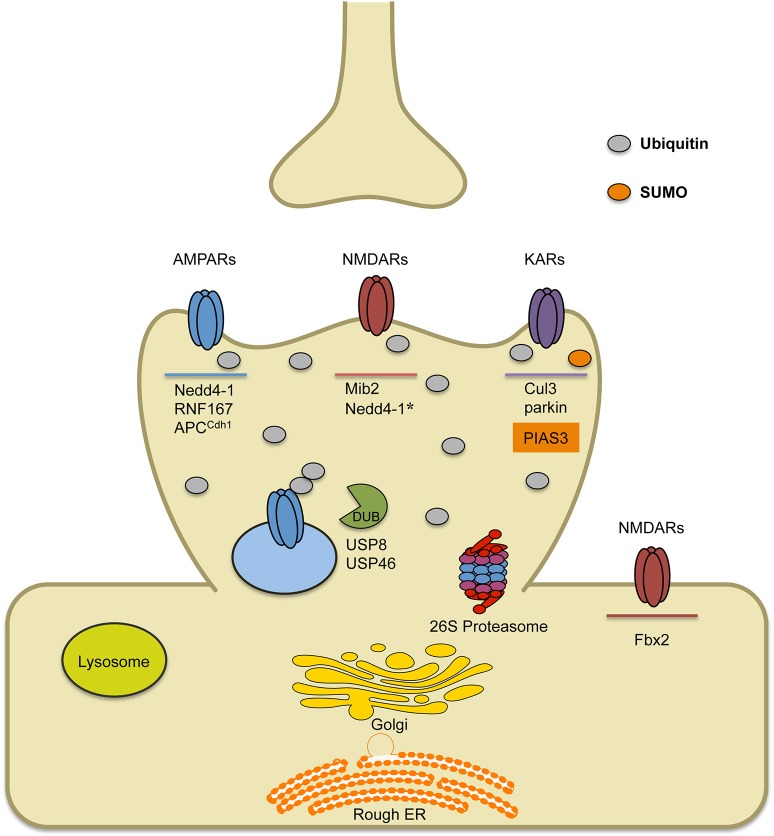
**Schematic of known E3 ligases and DUBs that target ionotropic glutamate receptors**. AMPARs are ubiquitinated by ligases Nedd4-1, RNF167 and APC^Cdh1^. Short-term treatment with bicuculline (on the order of min) leads to AMPAR ubiquitination by RNF167, while long-term treatment with bicuculline (on the order of hours to days) leads to AMPAR ubiquitination by either Nedd4-1 or APC^Cdh1^. Once internalized, ubiquitinated AMPARs can either be deubiquitinated by DUBs (USP8 or USP46), which can promote their recycling, or they can be targeted to the lysosome or the 26S proteasome for degradation. Ubiquitination of NMDARs occurs by the E3 ligase Mind bomb-2 (Mib2) and is, in part, regulated by receptor phosphorylation. In heterologous cells involving transfection strategies, Nedd4-1 has been shown to target NMDARs for ubiquitination, though data in neurons has not been verified. Furthermore, retrotranslocated NMDARs can be ubiquitinated by F-box Protein 2 E3 ligase, Fbx2, which recognizes high-mannose glycans found on the extracellular region of GluN subunits. Finally, KARs have been found to be ubiquitinated by Cul3 (through interactions with actinfilin) or parkin, which has been implicated in Parkinson's disease. Alternatively, covalent modification of KARs by the SUMO conjugating enzyme PIAS3, has been shown to regulate kainate-receptor mediated synaptic transmission.

Since the first investigations of glutamate receptor ubiquitination in the nematode Caenorhabditis elegans (*C. elegans)* (Burbea et al., 2002) and later in mammals (Schwarz et al., 2010; Fu et al., 2011; Lin et al., 2011; Lussier et al., 2011), more recent studies have further defined the role of ubiquitination on glutamate receptor trafficking and function (See Figure 1). In this review we will highlight recent findings on the ubiquitin-dependent trafficking and turnover of glutamate receptors in neurons and the distinct regulatory signals involved.

### Signals that induce AMPAR ubiquitination

Surface AMPARs are internalized in a constitutive manner, but their trafficking can also be controlled through synaptic activity (Huganir and Nicoll, 2013). Activation of glutamate receptors with the agonists AMPA and NMDA can both induce receptor internalization through independent pathways that result in different receptor fates (Shepherd and Huganir, 2007). Studies in recent years have shown that certain types of stimulation can induce AMPAR internalization through a pathway that involves receptor ubiquitination. In this section, we discuss what is currently known about the synaptic cues that induce AMPAR ubiquitination.

The first study of mammalian AMPAR ubiquitination determined that direct activation of receptors with the agonist AMPA causes robust ubiquitination of the GluA1 subunit (Schwarz et al., 2010), a finding which has been confirmed in recent studies (Scudder et al., 2014; Widagdo et al., 2015). Interestingly, another group found that AMPA promotes ubiquitination of the GluA2 subunit rather than GluA1 (Lussier et al., 2011). However, a recent report indicates that AMPA induces the ubiquitination of all four AMPAR subunits (Widagdo et al., 2015). Regardless of this discrepancy, all available data support the conclusion that direct activation of AMPARs with agonists promotes their ubiquitination and internalization. This ligand-induced effect requires calcium entry, provided mainly through voltage-gated calcium channels while NMDA receptor (NMDAR) signaling appears unnecessary (Schwarz et al., 2010; Lussier et al., 2011; Widagdo et al., 2015).

In addition to bath application of receptor agonist, many groups have used alternate methods to examine activity-induced AMPAR ubiquitination. The GABA_A_ receptor antagonist bicuculline is commonly used to globally raise activity in cultured neurons, and when applied to neurons for a prolonged amount of time can induce a negative feedback process termed synaptic scaling (Turrigiano et al., 1998; Siddoway et al., 2014). Bicuculline treatments have been shown to promote AMPAR ubiquitination after short-term and long-term treatments (Lussier et al., 2011; Scudder et al., 2014; Widagdo et al., 2015). Furthermore, long-term treatment promotes the recruitment of the E3 ligase Nedd4-1 to synapses (Scudder et al., 2014). However, unlike the AMPA-induced scenario, this form of receptor modification appears to require NMDAR signaling (Lussier et al., 2011; Widagdo et al., 2015), which suggests there may be slight differences in the pathways that involve ubiquitin conjugation. Application of an agonist activates both synaptic and extrasynaptic receptors while bicuculline should only activate synaptic AMPARs, and this difference may activate different cellular pathways and perhaps even lead to different receptor fates. Alternatively, these two scenarios may simply differ in the source of calcium; NMDARs could provide the calcium influx during bicuculline treatments while AMPA treatments instead rely on calcium influx through voltage-gated calcium channels and calcium-permeable AMPARs.

**Figure 2:**
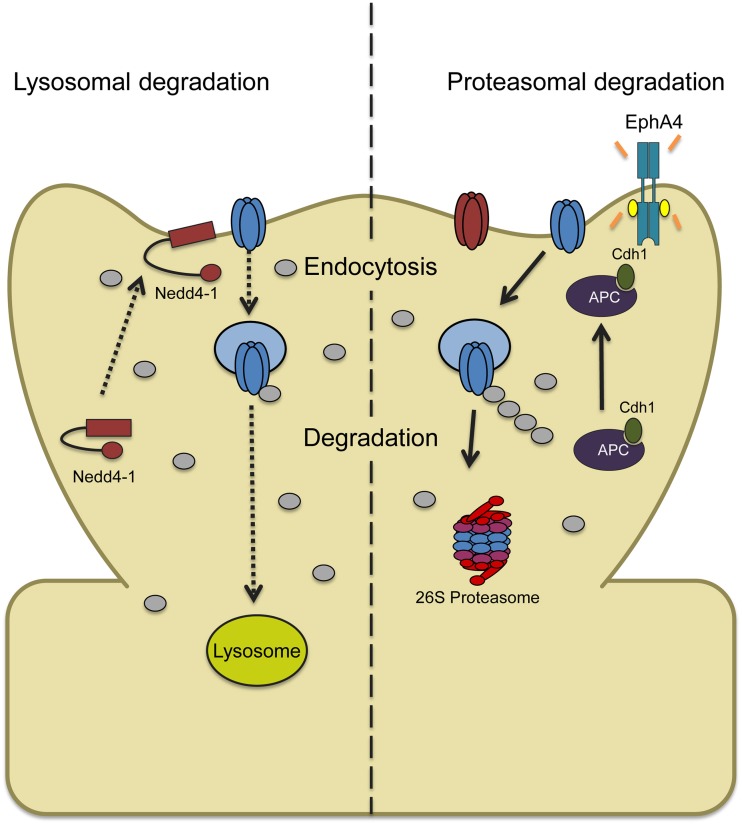
**Two models depicting ubiquitin-dependent AMPAR trafficking in synaptic downscaling. (Left)** Increased synaptic activity through prolonged bicuculline treatment leads to an increase and recruitment of the ligase Nedd4-1 at synapses with a concomitant decrease in the DUB USP8 levels. This causes a shift in the balance of AMPAR trafficking that overall favors Nedd4-1-dependent ubiquitination and internalization. Ubiquitinated AMPARs are then targeted to the lysosome for degradation. **(Right)** Bicuculline-induced downscaling can also be mediated by ubiquitin and proteasome-dependent degradation. Chronically elevating synaptic activity increases tyrosine kinase EphA4 activity which binds to the ligase APC and activator Cdh1, recruiting GluA1. APC^Cdh1^ then polyubiquitinates GluA1-containing AMPARs which targets them to the proteasome for degradation.

The specific ligase responsible for bicuculline-induced ubiquitination is debated; short treatments induce AMPAR ubiquitination that requires the ligase RNF167 (Lussier et al., 2012) while longer treatments (>20 h) recruit Nedd4-1 to synapses and increase the overall protein levels of Nedd4-1 (Scudder et al., 2014). The E3 ligase complex APC^Cdh1^ also appears to become engaged upon long-term bicuculline treatment, as loss of this protein prevents bicuculline's effects on synaptic strength, though it is unclear whether this is due to direct targeting of AMPARs (Fu et al., 2011). RNF167 may handle short-term regulation of surface AMPARs while Nedd4-1 and APC^Cdh1^ act on a longer time scale to homeostatically control synaptic strength, supported by the fact that bicuculline-induced synaptic scaling is blocked by the loss of either of these ligases (Fu et al., 2011; Scudder et al., 2014) (Figure 2).

In addition to pharmacological manipulations, AMPAR ubiquination has been studied using various other techniques. Using light-gated glutamate receptors to activate a subset of cultured neurons, Hou et al. demonstrated that synapses receiving 30 min of prolonged activity reduced their total and surface GluA1 and also experienced a site-dependent increase in polyubiquitin conjugates and the ligase Nedd4-1 (Hou et al., 2011). These data suggest that homeostatic scaling via ubiquitin-dependent pathways can occur on a single synapse level. To date, only a few groups have examined the role of receptor ubiquitination *in vivo* (Yuen et al., 2012; Atkin et al., 2015). Yuen et al. found that repeatedly exposing rats to stress leads to ubiquitination of GluA1 and the NMDAR subunit NR1 in the prefrontal cortex by Nedd4-1 and Fbx2, respectively, and that this results in reduced levels of these receptors and reduced glutamatergic transmission, which may underlie the stress-induced cognitive deficits observed.

### Function of ubiquitination in AMPAR trafficking

The field has converged on the idea that ubiquitination plays a critical role in regulating the abundance and localization of AMPARs in neurons. However, the exact role that ubiquitin conjugation plays remains debated. Direct conjugation to AMPAR subunits at the cell surface may function as a signal for internalization by triggering the assembly of endocytic machinery. Alternatively, the internalization process may occur prior to conjugation, with ubiquitination instead serving to direct endocytosed receptors toward a fate of degradation and prevent them from recycling to the surface.

In *C. elegans* it was first observed that the abundance of GLR-1, the *C. elegans* non-NMDA type glutamate receptor, is regulated by ubiquitin (Burbea et al., 2002; Juo and Kaplan, 2004). GLR-1 was found to be ubiquitinated *in vivo*, and mutations in GLR-1 which block ubiquitination increase the abundance of the receptor at synapses and alter locomotion behavior in a manner consistent with increased synaptic strength. In this system, overexpression of ubiquitin caused a decrease in GLR-1 abundance, and mutations in unc-11, which encodes the clathrin adaptin protein AP180, blocked the effect. Additionally, ubiquitin-conjugated GLR-1 accumulated in neurons lacking functional AP180. While the authors acknowledged that it is possible that ubiquitination is occurring in endosomes to control degradation, their data strongly supports a model where ubiquitination of GLR-1 occurs at the surface, prior to internalization through clathrin-mediated endocytosis (Burbea et al., 2002).

Three of the first papers to study mammalian AMPAR ubiquitination argued in favor of ubiquitination at the postsynaptic membrane (Schwarz et al., 2010; Fu et al., 2011; Lin et al., 2011). In these these studies, blocking ubiquitination by mutating the relevant GluA1 lysines (GluA1-4KR) or knocking down the E3 ligase responsible (Nedd4-1 or Cdh1, activator of APC) prevented the detection of internalized GluA1 during a stimulation-induced internalization assay. Thus, the authors concluded that ubiquitination of AMPAR subunits is a necessary step in the internalization of stimulated receptors. However, an alternate explanation could be that surface receptors are indeed internalized in these conditions, but upon failure of ubiquitination in a nascent endosomal vesicle, receptors are recycled back to the membrane during the time-frame of the internalization assay. In that case, the lack of a sorting signal by ubiquitin would cause the same observed effect as the lack of internalization. However, in agreement with the aforementioned studies, a recent paper identified a role for the endocytic adaptor protein Eps15, which is known to be critical in supporting the internalization of epidermal growth factor receptor (Goh and Sorkin, 2013), in ubiquitin-dependent receptor trafficking (Lin and Man, 2014). The authors found that levels of Eps15 affected surface expression of GluA1 through ubiquitin-dependent interactions with this subunit and also demonstrated that clathrin-mediated endocytosis is necessary for the ubiquitin-induced enhancement in receptor internalization (Lin and Man, 2014). Since Eps15 is involved in the recruitment of endocytic machinery at the surface, the authors conclude that this ubiquitin-mediated interaction is occurring prior to AMPAR internalization.

There have been studies of AMPAR ubiquitination which concluded that ubiquitination is not necessary for the internalization step of this pathway (Lussier et al., 2011; Widagdo et al., 2015). In investigating activity-induced GluA2 ubiquitination, Lussier et al. utilized dynasore to block dynamin-mediated endocytosis and sucrose to prevent formation of clathrin-coated pits and observed that these manipulations prevent the detection of bicuculline-induced ubiquitination of GluA2 (Lussier et al., 2011). This supports a model where ubiquitin conjugation occurs after internalization to control receptor sorting. Similarly, a recent paper observed that inhibition of dynamin-mediated endocytosis with dynasore abolishes bicuculline- or AMPA-induced ubiquitination of all four AMPAR subunits (Widagdo et al., 2015). Curiously, this study also utilized GluA1 mutants that cannot be ubiquitinated but found that these lysine mutations did not prevent agonist-induced internalization, in contrast to the previously described papers (Schwarz et al., 2010; Lin et al., 2011). Instead, the lysine mutations reduced the amount of internalized GluA1 that co-localized with LAMP-1 positive late endosome/lysosomes and allowed more GluA1 to return to the surface. As a result, the authors conclude that ubiquitination occurs after receptors have been internalized, likely in early endosomes.

While the role of ubiquitination in regulating AMPARs has only been explored fairly recently, extensive work has been done to identify the role of ubiquitin in controlling surface proteins in non-neuronal cells. Epidermal Growth Factor Receptor (EGFR) has been the subject of numerous studies, as its ligand-induced removal from the cell surface is regulated by the E3 ubiquitin ligase c-Cbl (Goh and Sorkin, 2013). Though considerable debate continues to exist surrounding the various internalization pathways, ample evidence has shown that direct ubiquitination of EGFR by c-Cbl can serve as a signal for the assembly of clathrin-mediated endocytic machinery (including Eps15) and subsequent endocytosis (Stang et al., 2000; de Melker et al., 2001). However, EGFR can also be internalized through a non-ubiquitin-dependent pathway, and ubiquitination can instead occur while EGFR is located in endosomes, where it serves as a signal for degradation (Levkowitz et al., 1998; Huang et al., 2007; Goh and Sorkin, 2013). Thus, it is reasonable that AMPAR internalization could occur through multiple pathways that involve ubiquitin, and that ubiquitination can occur on the surface to signal internalization or at early or late endosomes to control receptor fate. The exact conditions (i.e., type and intensity of neuronal stimulation) could determine which pathway surface AMPARs engage in. Since there appear to be a few distinct E3 ligases that can target AMPAR subunits, these ligases may engage the receptors at different points in the internalization process. Additionally, since AMPARs can be composed of four different subunits, the exact composition of surface receptors may also be a factor.

### Fate of internalized AMPARs

Though there is still uncertainty about the exact function of AMPAR ubiquitination, the ultimate fate of ubiquitinated AMPARs can be determined by the ubiquitin chain length and topology as well as the duration of the modification. Mono- and short-chain ubiquitination of membrane proteins often leads to their internalization and degradation by the lysosome, while ubiquitin chain lengths of four or more (polyubiquitination) typically targets substrates for proteasome-dependent degradation (Clague and Urbé, 2010). The duration and dynamics of the ubiquitinated state are, in many cases, regulated by other post-translational modifications and the activity of DUBs which counteract the ubiquitin conjugation forward reaction (Pickart, 2004). In the following sections we discuss evidence for both lysosomal and proteasomal degradation of AMPARs.

#### Lysosomal degradation of AMPARs

The lysosome is a membrane-bound organelle which contains hydrolytic enzymes that break down cellular components and allow them to be recycled. It maintains a low pH (pH < 5) through proton pumps nested inside the lysosomal membrane, which provides an ideal environment for the hydrolytic enzymes to function. Two main degradative pathways converge at the lysosome: the ESCRT pathway and the autophagy pathway. In the ESCRT pathway, membrane proteins are endocytosed and routed to the MVB and then to the lysosome (Hurley, 2008; Henne et al., 2011). In the autophagy pathway, cytoplasmic components are engulfed in an autophagosome, which fuses with the lysosome to form an autolysosome (Shintani and Klionsky, 2004; Levine and Kroemer, 2008).

Trafficking of AMPARs to the lysosome was first characterized in work by Ehlers MD, where he showed that bath application of AMPA targets AMPARs to the early endosome and subsequently to late endosome/lysosome compartments. Furthermore, AMPA-induced degradation was blocked by lysosomal inhibition (Ehlers, 2000). On the other hand, other studies have shown AMPAR subunit composition controls its trafficking. For instance, ectopic expression of tagged GluA subunits in cultured hippocampal neurons, which favors homomeric assembly, revealed that specific synaptic cues govern internalization and endocytic sorting to recycling or degradation pathways (Lee et al., 2004), while subunit-specific interactions with stargazin and PKC may control endocytic sorting to lysosomes (Kessels et al., 2009). The distinct trafficking of AMPARs based on subunit composition highlights the cell's ability to fine-tune surface proteins in order to tightly control changes in synaptic plasticity.

The large majority of AMPAR trafficking studies have revealed that the phospho-status of carboxy terminal residues tightly controls the stability of AMPARs at the synaptic membrane (Shepherd and Huganir, 2007). Our group, however, was the first to demonstrate that activity-dependent AMPAR ubiquitination by the E3 ligase Nedd4-1 targets AMPARs to the lysosome for degradation (Schwarz et al., 2010). Inhibition of the lysosome not only prevented AMPA-induced degradation of ubiquitinated GluA1-containing AMPARs but it also increased colocalization of AMPARs with lysosomes when Nedd4-1 was overexpressed (Schwarz et al., 2010).

One form of synaptic plasticity in which AMPARs are internalized and potentially degraded by the lysosome is long-term depression (LTD). Interestingly, however, one group found that inhibition of the lysosome did not affect LTD induction. Rather, expression of a dominant negative Rab7, which regulates trafficking from the late endosome to the lysosome, significantly reduced LTD expression compared to controls (Fernández-Monreal et al., 2012). The authors suggest that the sorting decision of internalized AMPARs between Rab7- or Rab11- dependent trafficking (which route to lysosome or back to synaptic membrane, respectively) is a key determinant for LTD induction. The authors also show that dephosphorylation of S845 on GluA1 is correlated with AMPAR degradation by the lysosome (Fernández-Monreal et al., 2012). Indeed, in GluA1 S845A phosphomutant mice, LTD is altered and AMPARs are constitutively degraded by the lysosome (He et al., 2009). This study supports the idea that LTD involves AMPAR internalization and degradation by the lysosome. However, it is still to be determined if AMPAR ubiquitination is required for LTD.

Recently, however, autophagy-dependent degradation of AMPARs has been shown to occur during LTD. Shehata et al. discovered that chemical LTD (chemLTD) induces autophagy-dependent degradation of AMPARs via inhibition of the PI3K-Akt-mTOR pathway (Shehata et al., 2012). Furthermore, they indicate that autophagosomes can enter dendritic spines in an activity-dependent manner, suggesting autophagy can degrade AMPARs (Shehata et al., 2012). One interesting avenue of research would be to explore lysosomal trafficking during synaptic plasticity and examine how lysosomal trafficking may change during these activity manipulations.

#### Proteasomal degradation of AMPARs

The ubiquitin proteasome system (UPS) is one of the most widely studied pathways for protein degradation in eukaryotic cells. Polyubiquitinated proteins are recognized by the 26S proteasome where they can be degraded into small peptides and amino acids. The 26S proteasome is a large energy-dependent protease formed by the co-assembly of a 20S proteasome (the catalytic component) and 19S cap (regulatory particle which binds ubiquitinated proteins) (Hershko and Ciechanover, 1998). It was first demonstrated by Zhang et al. that AMPAR turnover was proteasome-dependent. In this study, the authors showed that Na,K-ATPase (NKA) inhibition led to rapid degradation of AMPAR subunits which was blocked by proteasome inhibitors (Zhang et al., 2009). Additionally, they showed that AMPAR degradation is sodium-dependent during NKA inhibition. They reasoned that since Nedd4-1, an E3 ligase demonstrated by our lab to target AMPAR for ubiquitination (Schwarz et al., 2010), is also regulated by sodium, it is likely the ligase that ubiquitinates AMPARs and targets them for proteasome-dependent in response to NKA inhibition (Zhang et al., 2009). A follow-up study from this group found Nedd4-1 does indeed ubiquitinate AMPARs and that under basal conditions, inhibition of the proteasome leads to a build-up of ubiquitinated AMPARs (Lin et al., 2011). In addition, Hou et al. used light-controlled activity stimulation of synapses and found that AMPARs are degraded after repeated stimulation and inhibiting the proteasome prevented this loss, while lysosomal inhibition has no effect (Hou et al., 2011). While the observation of proteasome-dependent turnover of AMPARs differs from findings by our group, which showed that AMPAR activation leads to Nedd4-1-dependent ubiquitination and degradation of AMPARs by the lysosome (Schwarz et al., 2010; Scudder et al., 2014), it suggests there are multiple signaling pathways that can control turnover of AMPARs. Regardless, given how AMPAR trafficking and degradation must be tightly regulated it is not surprising that AMPARs can be degraded by both machineries.

#### Deubiquitination of AMPARs and recycling

While the ubiquitin signal can have a profound cellular effect, in some cases ubiquitinated proteins are spared from degradation. The ubiquitination process can be counteracted by DUBs, which remove the ubiquitin moiety. For membrane proteins such as AMPARs, deubiquitination can facilitate their recycling to the surface of the cell. There are 5 major classes of DUBs and they can function to cleave ubiquitin-linked molecules to (1) maintain the ubiquitin pool, (2) rescue proteins targeted for degradation, or (3) prevent UPS-dependent protein degradation. Two DUBs have been implicated in AMPAR deubiquitination: USP8 and USP46. USP8, which is found in the somatic, dendritic and synaptic compartments of neurons, becomes dephosphorylated and activated upon calcium influx (Scudder et al., 2014). Our group found that NMDAR activation negatively regulates AMPAR ubiquitination, suggesting that the influx of calcium through NMDAR channels activates USP8. This causes the deubiquitination of AMPARs, resulting in their ability to escape degradation and recycle back to the membrane. The functional importance of USP8 was further demonstrated when overexpression of USP8 prevented bicuculline-induced downscaling (Scudder et al., 2014). Since USP8 counteracts Nedd4-1's ability to ubiquitinate and target AMPARs for degradation, this study provides the first mechanistic evidence for opposing activity-dependent control of a ubiquitin ligase and DUB in the regulation of homeostatic plasticity.

USP46 has also been implicated in AMPAR deubiquitination. In the ventral nerve cord of *C. elegans* it was found that USP46 binds to GLR-1, and negatively regulates the levels of its ubiquitination. Conversely, mutant USP46 increases ubiquitinated GLR-1 (Kowalski et al., 2011). Mechanistically, USP46 can bind with WD40-repeat (WDR) proteins WDR-20 and WDR-48 to stimulate USP46 catalytic activity and increase GLR-1 levels (Dahlberg and Juo, 2014). Recently, in dissociated rat neuronal cultures, USP46 was found to deubiquitinate AMPARs (Huo et al., 2015). It appears that both USP8 or USP46 knockdown lead to elevated AMPAR ubiquitination and reduced miniature excitatory postsynaptic currents (mEPSC) amplitude while overexpression of either DUB leads to a reduction in AMPAR ubiquitination and an increase in surface AMPAR abundance (Scudder et al., 2014; Huo et al., 2015). Given that multiple E3 ubiquitin ligases and DUBs have been shown to target AMPARs, it will be of great interest to understand how the dynamics of AMPAR ubiquitination and deubiquitination are regulated or influenced by other post-translational modifications such as phosphorylation.

### Ubiquitination and SUMOylation of non-AMPA glutamate receptors

While the trafficking of AMPARs to and from the synapse is thought to underlie most changes in synaptic strength at excitatory synapses, control of other glutamate receptors is also critical in regulating transmission and the capacity for plasticity. The number of NMDA and kainate receptors (KARs) at the postsynaptic membrane can be controlled by multiple mechanisms, including direct ubiquitination. In this section we review what is currently known about ubiquitination and ubiquitin-like modification of NMDARs and KARs.

Like AMPARs, kainate receptors are internalized through separate pathways in response to NMDA treatment or direct activation by an agonist (kainate). Activation of NMDARs promotes the targeting of internalized KARs to recycling endosomes, allowing them to return to the surface, while direct activation of KARs causes the majority of internalized receptors to be degraded via lysosomes, thus reducing surface and total levels (Martin and Henley, 2004). Kainate-evoked endocytosis requires phosphorylation of the GluK2 subunit by protein kinase C (PKC) and the conjugation of the small ubiquitin-like modifier SUMO-1 by the SUMO conjugating enzyme PIAS3 (Martin et al., 2007). Treatment with kainate causes phosphorylation of C-terminal sites of GluK2, which then induces the SUMOylation of this region and causes the subsequent internalization and degradation of these receptors (Konopacki et al., 2011). SUMOylation is thought to occur at surface receptors and serve as a signal for endocytosis, as non-SUMOylatable GluK2 does not undergo agonist-induced endocytosis and kainate-induced SUMOylation of surface GluK2 is detected when internalization is blocked by sucrose (Martin et al., 2007). These studies indicate that SUMOylation serves as a critical signal to control surface expression of KARs and KAR-mediated synaptic transmission. The authors theorize that this mechanism may exist to protect neurons from excitotoxic damage. Additionally, one recent report indicates that SUMOylation of GluK2 is necessary for the long-term depression of KAR-mediated synaptic transmission evoked by low-frequency stimulation at mossy fiber-CA3 synapses, demonstrating a role for SUMO conjugation in activity-dependent synaptic plasticity (Chamberlain et al., 2012).

Kainate receptors can also be ubiquitinated by the E3 ligase parkin and the Cul3-containing E3 ligase complex (Salinas et al., 2006; Helton et al., 2008). GluK2 interacts with the postsynaptically-located protein actinfilin, which serves as a scaffold to bring the receptor subunit in contact with Cul3. Reduction of Cul3 or actinfilin leads to increased surface GluK2 and reduced ubiquitination of this subunit (Salinas et al., 2006). However, it is not yet known whether this occurs at surface KARs or whether GluK2 is ubiquitinated and degraded via an endoplasmic reticulum-associated degradation (ERAD) pathway. Since the neuronal activity-dependence of this phenomenon was not explored, this mechanism may constitutively control KAR levels. A recent study has also reported GluK2 ubiquitination, in this case by the E3 ligase parkin, a protein known to be mutated in many cases of Parkinson's disease (Maraschi et al., 2014). Parkin mutations in mice and human patients cause large increases in total levels of GluK2. Parkin appears to directly ubiquitinate this subunit and control its surface expression in neurons, and the interaction between these proteins increases after treatment with glutamate. As reported in other studies, loss of parkin increases the susceptibility of neurons to excitotoxic damage and death after treatment with kainate (Staropoli et al., 2003; Helton et al., 2008). Thus, the authors conclude that this ubiquitination pathway serves to protect neurons from excitotoxic damage and loss of this pathway through parkin mutations may contribute to the pathology of Parkinson's disease. Collectively, these studies indicate that a combination of phosphorylation, ubiquitination, and SUMOylation work to control KAR surface abundance and allow for synaptic plasticity and protection from excitotoxic stress.

Control of surface NMDARs is critical in regulating synaptic transmission and synaptic plasticity, and also in limiting excitotoxicity. In response to prolonged increases in activity caused by bicuculline *in vitro*, the subunit GluN1 was found to be ubiquitinated by the E3 ligase Fbx2 and the synaptic levels of this subunit are reduced, suggesting that ubiquitination may serve as a mechanism to reduce receptor levels during synaptic scaling (Kato et al., 2005). However, ubiquitin conjugation occurs at an extracellular domain of GluN1, through a mechanism involving retrotranslocation of NMDARs. Ubiquitination of GluN1 by Fbx2 was also reported to occur *in vivo* in the prefrontal cortex as a result of repeated stress. This mechanism appears to partially underlie stress-induced cognitive impairments, in conjunction with GluA1 ubiquitination by Nedd4-1 (Yuen et al., 2012). Recent studies from *Fbx2* knockout mice showed increases in GluN1 and GluN2A but no changes to GluN2B levels and that the increased GluN1 subunits are mostly found at the cell surface. The build-up of unused NMDAR subunits results in an accumulation at non-synaptic sites leading to the formation of shaft synapses (Atkin et al., 2015). It was found that high-mannose glycans reside on the extracellular region of GluN subunits and that Fbx2 can bind to high-mannose glycans (Atkin et al., 2015). This suggests that internalization must precede Fbx2-directed ubiquitination of GluN subunits.

Ubiquitination of the subunit GluN2B by the ubiquitin ligase Mind bomb-2 (Mib2) has also been reported (Jurd et al., 2008). In this pathway, phosphorylation of this subunit causes direct ubiquitination and downregulation of surface NMDARs, potentially to prevent the pathological effects of excessive NMDAR activation. Nedd4-1 has also recently been reported to ubiquitinate the GluN2D subunit and decrease NMDAR signaling, though this has not yet been verified in neurons (Gautam et al., 2013) (See Figure 1, Nedd4-1^*^). Taken together, these studies indicate that the surface expression of NMDARs is tightly regulated by many mechanisms, several of which involve direct ubiquitin conjugation to receptor subunits. These pathways likely work to both homeostatically control surface expression and protect neurons from excitotoxic stress.

### Degradation of glutamate receptor interacting and postsynaptic scaffold proteins

The trafficking of glutamate receptors to and from the postsynaptic membrane in part relies on direct and indirect interactions with other proteins and these interacting proteins can regulate many forms of plasticity at excitatory synapses. Several of these proteins act as scaffolds or regulatory proteins to ensure the proper postsynaptic insertion, removal, or stabilization of glutamate receptors. As such, the ubiquitin-dependent degradation of these proteins could therefore have profound effects on glutamate receptor trafficking and function as well as synaptic plasticity.

One of the first studies that examined protein turnover at synapses revealed that the ubiquitination and degradation of several PSD proteins was regulated by synaptic activity. Interestingly, these effects were controlled by chronic activity modulation and found to be bi-directional. The turnover of key ionotropic and metabotropic glutamate receptor scaffolding molecules including Shank, AKAP79/150 (AKAP), and GKAP was found to be mediated by UPS-dependent degradation (Ehlers, 2003). Subsequently, the E3 ubiquitin ligase TRIM3 was identified to target GKAP (also known as SAPAP) for ubiquitin-dependent degradation (Hung et al., 2010). Furthermore, activity- and phosphorylation-dependent ubiquitination and degradation of GKAP was shown to be important for global remodeling of synapses. Altering GKAP levels at synapses by overexpression or knockdown alters the remodeling of PSD-95 and Shank and blocks bidirectional synaptic scaling (Shin et al., 2012). This indicates that half-life control of specific PSD scaffolds and regulatory proteins is important for the overall activity-dependent remodeling of synapses.

The ubiquitination of PSD-95, a major PSD scaffold that links both NMDA- and AMPA-type glutamate receptors to signaling complexes and to the actin cytoskeleton (Kim and Sheng, 2004), has been reported by several groups. Colledge et al. found PSD-95 to be ubiquitinated by the E3 ligase Mdm2 in response to NMDA receptor activation. Furthermore, they showed that preventing PSD-95 ubiquitination and degradation blocked NMDA-induced AMPAR internalization and synaptically-induced LTD (Colledge et al., 2003). In contrast, Bianchetta et al. found that increased cyclin-dependent kinase 5 (Cdk5) activity promotes PSD-95 ubiquitination by increasing Mdm2 association with PSD-95. In this case, however, they found that PSD-95 levels were unchanged (Bianchetta et al., 2011). The authors therefore proposed a non-proteolytic role for PSD-95 ubiquitination involving increased interaction with the clathrin adaptor protein complex protein AP-2 to promote NMDAR-induced internalization of AMPARs (Bianchetta et al., 2011). More recently, the ubiquitination and degradation of PSD-95 has been linked to autism spectrum disorders (ASDs). Tsai et al. found that the myocyte enhancer factor 2 (MEF2) and fragile X mental retardation protein (FMRP)-regulated ASD-linked gene, protocadherin 10 (Pcdh10), links ubiquitinated PSD-95 to proteasomal turnover. In contrast, blocking Pcdh10 interaction with proteasomes prevented PSD-95 degradation and synapse elimination (Tsai et al., 2012).

Interestingly, negative regulators of synaptic AMPARs are also degraded by the UPS. Arc is an important synaptic protein that has been shown to promote the internalization of AMPARs (Chowdhury et al., 2006; Shepherd et al., 2006). While investigating the function of *Ube3A*, the gene mutated in the neurological disorder Angelman syndrome, Greer et al. found that loss of *Ube3A* prevents Arc ubiquitination and degradation with a concomitant decrease in AMPARs (Greer et al., 2010). It has been shown, however, that Ube3A may regulate Arc protein levels independent of direct ubiquitination (Kühnle et al., 2013). More recently, Mabb et al. found that the RING domain ubiquitin ligase Triad3A/RNF216 targets Arc for ubiquitination and degradation. In the absence of Triad3A, Arc levels are increased, leading to a loss of AMPARs and disruption of Arc-dependent forms of synaptic plasticity (Mabb et al., 2014).

PICK1 and GRIP1, two other AMPAR interacting and scaffold proteins, have also been shown to be regulated by ubiquitin-dependent protein degradation. The E3 ligase parkin, encoded by a gene involved in Parkinson's disease, was found to target PICK1 (Joch et al., 2007). In this case, however, PICK1 was found to be mono-ubiquitinated by parkin, which negatively regulates acid-sensing ion channels (ASIC). Therefore, it is speculated that enhanced ASIC activity could promote neurodegeneration in Parkinson's disease (Joch et al., 2007). While yet to be determined, it is plausible that parkin-mediated ubiquitination of PICK1 could regulate its interaction with AMPARs. GRIP1, which is primarily complexed with GluA2-containing AMPA receptors stabilized at the postsynaptic membrane (Kim and Sheng, 2004), was found to be rapidly degraded in an activity and calcium-dependent manner. Proteasome inhibition blocked these effects, indicating GRIP1 turnover to be proteasome dependent. Advancements in ubiquitin proteomics, where diglycine affinity strategies are now being used to enrich substrates and identify sites of ubiquitination (Na et al., 2012), will inevitably uncover other synaptic proteins that regulate glutamate receptor trafficking, function, and synaptic plasticity.

## Conclusion

In recent years, glutamate receptor ubiquitination has emerged as a key post-translational modification that can control glutamate receptor trafficking and degradation. The discovery that glutamate receptors can be tagged by ubiquitin in an activity-dependent manner highlights its importance in modulating synaptic plasticity. Interestingly, while several studies have revealed that internalized receptors can be recycled back to the synaptic membrane, the ultimate degradative fate of the receptors has been far less studied. In this review we discussed ubiquitination as a signal for glutamate receptor degradation by the lysosome or the proteasome. Available data suggests that glutamate receptors can be degraded by these cellular components but detailed mechanisms for their trafficking have not been fully elucidated and would be a particularly interesting area of research. Additionally, other post-translational modifications such as phosphorylation have also been shown to play a role in glutamate receptor trafficking. Since the phosphorylation status of AMPAR subunits is a key determinant of their synaptic abundance, it remains to be determined how phosphorylation and ubiquitination of glutamate receptors are coordinated. It may be that degradation of glutamate receptors is regulated by the dynamic interplay between receptor phosphorylation and ubiquitination. Pursuing these questions would ultimately provide insight into how neurons regulate receptor trafficking and turnover with high specificity in response to signaling cues.

### Conflict of interest statement

The authors declare that the research was conducted in the absence of any commercial or financial relationships that could be construed as a potential conflict of interest.
